# Public Perceptions of Treating Opioid Use Disorder With Deep Brain Stimulation: Comment Analysis Study

**DOI:** 10.2196/49924

**Published:** 2024-08-16

**Authors:** Patricia Henegan, Jack Koczara, Robyn Bluhm, Laura Y Cabrera

**Affiliations:** 1 Department of Engineering Science and Mechanics Pennsylvania State University University Park, PA United States; 2 College of Natural Sciences Michigan State University East Lansing, MI United States; 3 Department of Philosophy Michigan State University East Lansing, MI United States; 4 Lyman Briggs College Michigan State University East Lansing, MI United States; 5 Rock Ethics Institute Pennsylvania State University University Park, PA United States

**Keywords:** deep brain stimulation, DBS, comment analysis, refractory opioid use disorder, substance abuse, opioid addiction, opioid, substance use, opioid use, treatment, addiction, mental health, therapeutic, psychiatric disorder

## Abstract

**Background:**

The number of opioid-related deaths in the United States has more than tripled over the past 7 years, with a steep increase beginning at the same time as the COVID-19 pandemic. There is an urgent need for novel treatment options that can help alleviate the individual and social effects of refractory opioid use disorder (OUD). Deep brain stimulation (DBS), an intervention that involves implanting electrodes in the brain to deliver electrical impulses, is one potential treatment. Currently in clinical trials for many psychiatric conditions, including OUD, DBS’s use for psychiatric indications is not without controversy. Several studies have examined ethical issues raised by using DBS to counter treatment-resistant depression, obsessive-compulsive disorder, and eating disorders. In contrast, there has been limited literature regarding the use of DBS for OUD.

**Objective:**

This study aims to gain empirical neuroethical insights into public perceptions regarding the use of DBS for OUD, specifically via the analysis of web-based comments on news media stories about the topic.

**Methods:**

Qualitative thematic content analysis was performed on 2 Washington Post newspaper stories that described a case of DBS being used to treat OUD. A total of 292 comments were included in the analysis, 146 comments from each story, to identify predominant themes raised by commenters.

**Results:**

Predominant themes raised by commenters across the 2 samples included the hopes and expectations with treatment outcomes, whether addiction is a mental health disorder, and issues related to resource allocation. Controversial comments regarding DBS as a treatment method for OUD seemingly decreased when comparing the first printed newspaper story to the second. In comparison, the number of comments relating to therapeutic need increased over time.

**Conclusions:**

The general public’s perspectives on DBS as a treatment method for OUD elucidated themes via this qualitative thematic content analysis that include overarching sociopolitical issues, positions on the use of technology, and technological and scientific issues. A better understanding of the public perceptions around the use of DBS for OUD can help address misinformation and misperceptions about the use of DBS for OUD, and identify similarities and differences regarding ethical concerns when DBS is used specifically for OUD compared to other psychiatric disorders.

## Introduction

As early as the late 1950s, lobbyists began pursuing pain medication legislation throughout the United States [1-3]. Based on the idea that chronic pain should be treated by physicians, US laws included the lack of prosecution of physicians prescribing pain medications in the 1980s [1,2]. In the United States, the prescription of opioids to provide pain relief has been debated since the mid-1990s, when the US Food and Drug Administration (FDA) approved oxycodone as a chronic pain medication, after which the overprescription of opioids and subsequent substance abuse became more prevalent [4]. There is evidence that the number of overdose deaths per year has increased significantly since 2010 [5], with a 3-fold increase in the United States between 2015 and 2021 [6,7], specifically affecting those in lower socioeconomic status regions of the United States [8-12]. Many countries, including the United States, recognize the psychological, social, and economic burdens of opioid addiction [13,14]. Several of these countries have implemented preventative measures, like prescription restrictions and prescription drug monitoring programs, to minimize refractory opioid use disorder (OUD) [15,16] mortality rates [17,18].

OUD is defined by the *DSM-5* (*Diagnostic and Statistical Manual of Mental Disorders* [Fifth Edition]), as a compulsive dependence on opioid consumption that negatively impacts a person’s life [15,16]. OUD is considered to be refractory when patients do not show a decrease in drug use after being treated by all FDA-approved treatments including buprenorphine, methadone, behavioral counseling, and behavioral interventions [15,16].

Deep brain stimulation (DBS) has been proposed as a potential treatment for refractory OUD [19-21]. DBS involves the implantation of electrodes into the brain to regulate abnormal neural activity. It is FDA-approved for various movement disorders, such as Parkinson disease, and under investigation for psychiatric conditions [22,23]. There are currently 8 clinical trials listed in the US National Institutes of Health’s clinical trial database focused on “opioid addiction” (the term used by international studies [21]) or “OUD” (US studies [24]), including the pilot study discussed in the news stories we address here.

The use of DBS for psychiatric conditions raises ethical considerations including concerns about informed consent [25], treatment safety [26], threats to patient agency [27], and the treatment being too intrusive [28,29]. Ethical discussion of the use of DBS for addiction [30] must also consider the stigmatization and criminalization of OUD [31]. While a number of empirical studies assess public perceptions of the use of DBS for psychiatric disorders [28,29,32], ours is the first, to our knowledge, to focus on public perceptions of DBS to treat OUD. The analysis of reader comments on web-based media stories offers valuable insights into public opinions and the factors that shape these opinions [33]. A better understanding of these perceptions can help address misinformation and misperceptions about the use of DBS and OUD [34,35]. This paper aims to provide insight into public perceptions of and concerns about using DBS to treat OUD, using public comments on web-based news stories about this therapy.

## Methods

### Source of Content Analysis

We used qualitative thematic content analysis [36,37] to examine and compare public comments on 2 Washington Post stories that described the case of an individual who entered a DBS pilot study for refractory OUD [38,39]. The first story [38] was published in 2019, directly preceding the procedure, while the second story [39] followed up with the same study participant’s results in 2021. The second story reported that the patient had been in sustained remission for over 600 days post implantation [39]. To our knowledge, these are the only news stories in major news outlets focusing on DBS and OUD that have been published with an extensive number of public comments. Our queries to verify this information have included sources, such as BBC News [40], CNN [41], and local news stories [42-44], focusing on the US clinical trials. All queried stories did not have publicly available comments [45] directly below the stories.

### Ethical Considerations

Publicly available information used for commentary analysis is generally exempt from institutional review board (IRB) review because it involves data that are already accessible to the general public and does not involve direct interaction with or personal data from individuals. Since this type of information, including news stories, social media content, and public records, is disseminated openly and is not subject to privacy restrictions, it typically does not pose the ethical concerns that IRB reviews are designed to address. The data used in this study was deidentified. We did not apply for an ethics board review assessment, as according to a Common Rule exemption criteria, “research uses of identifiable private information” (including information found on the web) are exempt from typical Common Rule protections, such as full board or expedited IRB review if such information is “publicly available” without restriction (45 Code of Federal Regulations 46.104(4)(i)). However, it is essential to remain mindful of ethical considerations, such as ensuring that interpretations are accurate and that the use of such data adheres to principles of fairness and respect.

### Qualitative Analysis

Our analysis is based on 292 comments. This includes all 146 comments from the 2019 story. For the 2021 story, 146 (of a total of 579) comments were randomly selected to facilitate a comparison of the themes found in the two sets of comments and to avoid biasing the quantification of comments toward the 2021 story [46]. Excel (Microsoft Corp) was used to organize and code the data, as well as to randomize the comments included in the second story. Each comment was treated as a single unit of analysis, even when the same commenter had multiple comments for the same piece. Comments were coded according to the theme or themes they elicited. After curating the data set to eliminate “not relevant” comments, our final number of comments for the 2019 story was 101, and 80 comments for the 2021 story.

Comments coded as “not relevant” included comments not addressing DBS for OUD or OUD itself. For example, some comments only discussed topics related to Alcoholics Anonymous and were therefore considered off-topic.

An example of a comment that shows how a comment can be still “on-topic” but not directly relevant to our codebook’s themes.

GOP needs electrodes implanted to vote the correct way.

2019 story, ID 45

This comment showcases the Alcoholics Anonymous “sidebar conversation” that was prominent throughout our 2021 story sample.

My experience with 12 step programs is that they are not terribly helpful.

2021 story, ID 444

Our qualitative thematic analysis used the codebook adapted from a previous study, based on web-based public comments on stories about treatments for psychiatric conditions, which included DBS [46], with the addition of several novel themes that emerged during the analysis of this data set and that reflected concerns specific to DBS for OUD. Coding was conducted by 2 independent coders (RB and JK), using iterative review and agreement after the coders discussed differences in codes.

### Statistical Analysis

We used descriptive and inferential statistics to characterize the composition and properties of the samples. Data were analyzed using SPSS (version 26; IBM Corp). Major themes were defined as themes that had cumulative percentages of comments greater than 10%. The threshold of *P*<.05 was set for statistical significance for the comparison of comments across the 2 stories.

## Results

### Major Themes Across Both Stories

#### Overview

In what follows, we examine themes commonly appearing in both stories. The percentage

of comments for all themes in both stories is found in Table 1. Four themes showed no significant differences between the 2019 and 2021 stories, as displayed in Table 2.

**Table 1:** Comparison of the percentage of themes per relevant comments in Bernstein (2019) and Bernstein (2021).

Theme	Percent of coded theme per relevant comment, %	
	Bernstein (2019)	Bernstein (2021)
Controversial^a^	37	5
Overall Therapeutic Need^a^	10	30
Is Addiction a Mental Disorder?	14	24
Overall Social Issues^a^	8	18
Resource Allocation^a^	19	7
Personal Anecdote (Patient)^a^	9	13
Cautionary Realism^a^	17	4
Hopes and Expectations	9	11
Scientific Validity	18	1
Stigma	5	11
Sarcastic Humor	10	4
Medical Professional Issues^a^	3	11
Optimism	5	5
Direct Modification of the Brain	6	4
Questioning Effectiveness of the Intervention	0	10
Industry Related Issues	6	3
Risk/Safety	8	0
Pop Culture References	6	1
Historical Lesions	6	1
Overall Disadvantages	6	0

^a^*χ*^2^_1_ with a 2-tailed Fisher Exact test showed statistically significant results (*P*<.05) for relevant themed comments with cumulative percentages greater than 10% (Table 2).

**Table 2:** Results from χ^2^_1_ (N=382) with a 2-tailed Fisher Exact test for relevant themed comments with cumulative percentages greater than 10%.

Theme	Fisher Exact 2-tailed *P* value (comparing 2019 and 2021)
Controversial	<.001
Resource Allocation	.09
Scientific Validity	.02
Cautionary Realism	.02
Is Addiction a Mental Disorder?	.15
Overall Therapeutic Need	<.001
Personal Anecdote	.01
Hopes and Expectations	.48
Overall Social Issues	.04
Stigma	.18
Medical Professional Issues	.03

#### Resource Allocation

The financial cost of undergoing DBS surgery, together with considerations about insurance coverage, were concerns captured under this theme. The availability of both the treatment and of qualified physicians was described as a limited resource. A comparison of the cost of saved lives to the financial cost of getting DBS was made in a few of the comments in the 2019 story. Other comments discussed the question of who should pay the financial cost of getting DBS (eg, the general public, the creators of opioids, or the families of patients with OUD).

A comment that mentions that health insurance is needed to cover the cost of DBS.

I’m sure most have insurance to cover the cost of treating opioid addiction with brain surgery. That’s absurd.

2019 story, ID 4

This comment elaborates on how limited the number of health care providers and facilities are in different regions.

It was only recently that treatment options were more readily available (in some parts of the country) ... And still. It’s hard to find good help. Even in a place that doesn’t have a shortage of therapists, psychiatrists, and x-waivered prescribers (those who can prescribe buprenorphine) it’s difficult to find a qualified clinician.

2021 story, ID 45

#### Is Addiction a Mental Disorder?

Commenters disagreed about whether OUD should be considered a mental disorder. Several commenters expressed that addiction, in their view, is not a medical problem. Comments on whether addiction can be mitigated by self-control occurred for both stories but were more frequently made in the 2021 story, as shown in Table 1.

This comment illustrates the perspective that OUD should be considered a decision and not an illness.

It’s not a moral issue. Simply a matter of deciding what you want to do, then doing it. Battery powered Nirvana is not Nirvana. Nor is a marathon record made by driving a motorcycle, a real marathon record.

2019 story, ID 21

This comment shares the potential implications of categorizing addiction as a choice, instead of an illness.

Addiction is the result of disease. It’s not pretty, but as long as we consider it a social issue instead of a medical one, it will be ignored, stigmatized and improperly treated.

2021 story, ID 93

This comment includes the perspective that people with addiction have to want to get better.

I do believe addiction, for the most part, is a decision. Yes, treat it as a health issue, but one that the dependent person is willing to heal...

2021 story, ID 97

#### Hopes and Expectations

This theme captures comments expressing hope for positive outcomes for the research or for the specific patient discussed in both stories or a strong belief that DBS can be successful in the treatment of refractory OUD.

This comment shows a positive perspective on treatments evolving with OUD or addiction.

Hopefully, technology and medical techniques like this will help provide some relief for people like [the patient], who desperately want to get clean, but are being constantly and actively undermined by countless years of neurochemical evolution.

2019 story, ID 66

This comment cheers on the success of DBS in the 2021 story.

I mean it seems like one of many tools in this guy’s arsenal of sobriety. Either way, though, I am grateful for his success and his giving back to the community.

2021 story, ID 126

#### Stigma

Comments coded under this theme referred to the stigma associated with DBS as a potential treatment for OUD and with OUD itself. Using DBS as a treatment method for OUD was described as potentially treating one problem by creating another problem, electrical stimulation addiction replacing OUD. A handful of comments supported the destigmatization of those with OUD.

This flippant comment illustrates the perspective on DBS by suggesting that the user will become addicted to the electrical stimulation.

Oh, great ... now they’ll be addicted to electrodes.

2019 story, ID 34

This comment includes a bigger picture of how a person with addiction might behave with something other than substance abuse or OUD.

...Addicts don’t choose to be addicts. They suffer from an inability to delay gratification no matter how inconsequential the delay. The “addictive personality” can be spotted at a very young age. The addict will always choose an immediate reward even if delaying the reward by a mere ten seconds will earn them a double reward.

2021 story, ID 461

### Key Areas of Difference Between Stories’ Comments

In what follows, we examine statistically different themes. In particular, we found that 7 themes showed significant differences between the 2019 and 2021 stories.

#### Controversial

Comments were coded as controversial when they included snarky comments or sparked passionate discussion and disagreement. This theme had the largest difference in the percentage of coded comments between the two stories. There were significantly more comments coded in this theme for the 2019 story. Most comments on this theme blamed corporations, physicians, political associations, or the legal system for the prevalence of OUD.

This comment includes a discussion of the conflict innately in the rate of OUD’s potential original cause.

Most addicts probably do not get their drugs from official/legal sources. But Wow ... Did the “corporation” tie the person to a bed and force them to become addicts? Where is the inalienable right to make personal choice we generally hold dear as a fundamental freedom? Or are choices good ... only until we can blame someone else for the bad choices we make? Sue all chocolate factories for my cravins?

2019 story, ID 117

This comment blames health care providers for their part in the current rate of OUD in the United States.

At the time, doctors knew little about the opioid disaster they were unleashing, which has since claimed half a million lives. They didn’t know that heroin was addictive? Or they were just making too much money by getting people addicted to ask questions?”

2021 story, ID 171

#### Scientific Validity

This theme captures the mention of either questioning or asserting scientific evidence. For example, the mention of there not being enough research, issues regarding the premature use of DBS, or comments that question the effectiveness of intervention were coded under this theme. Comments often had a tone of disbelief toward the potential benefits of DBS, however, comments on the scientific process of testing novel treatments were also included.

This comment questions the effectiveness of DBS as a treatment for OUD.

A fix that’s too quick and easy to be true, likely is too quick and easy to be true.

2019 story, ID 21

This comment includes the rigor of clinical trials, such as the DBS trial for OUD.

You might also understand that there is no treatment without research testing and clinical trials. This is research.

2019 story, ID 61

This commenter questioned the validity of using DBS as a treatment for OUD.

This is medical quackery.

2021 story, ID 430

#### Cautionary Realism

This theme captures comments that see the potential promise of the treatment but give cautionary warnings for the future. Comments made by readers of the 2019 story expressed “Cautionary Realism” more than twice as frequently compared to the later story, as shown clearly in Table 1.

This comment includes positive and uncertainty toward DBS as a treatment method for OUD.

This is terrifying and exciting all at once. This guy is very brave to try this. I hope that it helps him stay sober, as he obviously wants to.

2019 story, ID 121

This comment gives credence to DBS as a treatment method, while including a sense of hesitancy regarding the number of times it has been implemented.

...The surgical implant route, while radical and infrequent, deserves the detailed exploration given in this article.

2021 story, ID 649

#### Therapeutic Need

Commenters frequently mentioned that there are few therapies available to persons with OUD, emphasizing the need for novel treatments in this space. The percentage of comments in this category was much larger for the 2021 story than for the 2019 story. Many comments discussed potential alternatives to DBS, which were not described as options in the stories themselves. Another topic included in the comments involved the variability of needs of different people with OUD.

This comment touches upon the resistant portion of refractory OUD when describing the lack of treatment options and therefore hope.

Amazing to turn to this type of drastic invasive treatment, unless it’s truly for those who have no other hope at all. And where is the discussion of psychedelic drugs, which have been showing the greatest benefit with the least down side?

2019 story, ID 140

This comment points out the variations of OUD and how given those variations, not all treatments work the same for all.

Addiction is insidious and no one size fits all approach has been discovered.

2021 story, ID 7

#### Personal Anecdote

Comments coded under this theme capture stories from commenters who have struggled with addiction and are sharing their story to sympathize with the story, or who have had DBS or similar interventions for another disorder, or commenters who are giving accounts of friends or family on their experiences with addiction. This theme contained significantly more comments on the 2021 story.

This comment includes the commenter’s somewhat surreal experience after receiving DBS.

Had DBS for a tremor disorder. Worked like a charm. I hope this is successful for this person. And yes, it’s pretty wild to be awake during brain surgery. We were all talking about a TV show we liked and then they wanted me to draw a spiral. Oh, so you've already put the wire in?

2019 story, ID 1

This comment includes an experience making the decision to not take prescribed opioid painkillers.

Before the awareness of the over-prescription of opioids, I have had doctors recommend Codeine and Oxycontin for various needs. They also said I could just try more milligrams of an over-the-counter painkiller to get by. I am so glad I always chose the later.

2021 story, ID 533

#### Overall Social Issues

This code was used for a variety of comments on social issues not captured by other codes covering social themes. Comparing OUD to other diseases, generational differences in perspective on DBS and OUD, government regulation of OUD and DBS, economic drivers, and access to health care were a few of the social issue topics described in these comments. More comments on the 2021 story were coded with this theme.

This comment focuses on society’s potential response to making opioids more available.

Perhaps your arguments would be effective for the supply side of the equation, but they do nothing to reduce the demand side. Legalizing it would mean that opioids are more available, and more people would become addicted.

2019 story, ID 80

This comment includes various other implications of changing behavior with the use of neurotechnology such as DBS.

If brain stimulation can be used to curb addictive impulses, then maybe it can also be used to alter other kinds of behavior as well. Maybe someday convicted criminals will be given the opportunity to allow implantation of the probes as an alternative to serving time in prison. Think of all the possible uses of such a technology!

2021 story, ID 216

#### Medical Professional Issues

These comments included any mention of professional inertia, physicians as gatekeepers to care, doctor-patient relationships, conflicts of interest, and the management of patient care. A higher percentage of comments were coded under this theme for the 2021 story.

This comment includes a negative perspective on health care providers and opioid prescription.

He knows more than you Mr. Handout for Treatment Money. The opiates ARE MEDICINE for SEVERE pain and TRUE addiction is RARE. 996 in 1000 new to an opiate WILL NEVER ADDICT. Good health to you, they are now almost completely unavailable in therapeutic doses from any Doctor of Medicine.

2019 story, ID 75

This comment includes a potential future policy to require more vigilante follow-up from health care providers prescribing opioids.

The solution is for the doctor to closely monitor opioids post-op and refuse to refill the prescription and move the patient to an alternative as soon as possible.

2021 story, ID 580

## Discussion

### Principal Results

Overall, our results indicate that members of the public regard the treatment of refractory OUD using DBS with both some optimism and some concern. In addition to discussing the therapy, a number of comments addressed OUD itself, making it clear that both the disorder and its treatment shape commenters’ views.

A number of themes were commonly raised in comments on both of the Washington Post stories, while others were common for only one of them. Here, we consider these common themes in the context of the broader discussion of the ethics of DBS. Where applicable, we also address the differences in comment frequency in light of the framing of each of the original stories.

When relatively new medical interventions, such as DBS, appear on the horizon for disorders that have had such a toll on families and society, there are “Hopes and Expectations” that arise surrounding them. Considering the social [47-49], psychological [34,43], and economic [11,18] toll of OUD, these public comments portraying hope and expectation might mirror the desire to find something that can finally help with the toll that OUD takes on individuals, their loved ones, and society. The prevalence of this theme may also be attributed to the expectation that the public has about the scientific community finding treatments for various conditions in an efficient manner [50], but also to the often optimistic way in which novel treatments are portrayed in the media. In Cabrera et al [46], “Optimism” was a broader theme that encapsulated many of the same ideas as “Hopes and Expectations.” Much like “Hopes and Expectations,” the theme “Optimism” was found in multiple stories regarding DBS. For example, DBS is sometimes described in an overly optimistic manner in scientific journal studies [51,52], and it has been noted that the tone of media coverage of DBS is becoming increasingly more positive as time goes on [53]. This is important because the media acts as an intermediary between clinical researchers and the public [54]. Therefore, if the media continues to portray DBS in an overly optimistic way, focusing primarily on its benefits rather than offering a more balanced account of its pros and cons, this can create further misunderstandings and unfounded hopes for the general public [52].

“Stigma,” another major theme raised in both stories’ comments, reinforces barriers to the implementation of evidence-based interventions to prevent opioid overdose deaths [31,34,55-57]. Stigma has 2 important components. There is a stigma around psychiatric disorders in general [29,45,55,58], as well as a particular stigma related to treating OUD with DBS [34,55,57,58]. For example, almost half of the news stories included in the 10-year analysis by McGinty et al [57] use language that depicts opioid use in a stigmatizing manner. Furthermore, the way some media stories frame those with OUD as criminals instead of people needing treatment [56,59], further stigmatizes those with OUD. The stigma associated with receiving treatment for OUD can also be found with regard to other addiction-related diseases, as measured by the Shatterproof Addiction Stigma Index [55,58]. The Shatterproof Addiction Stigma Index was created to help measure levels of stigma, and to help combat stigma, discrimination, and barriers to care associated with substance use disorder [55,58]. There is also a stigma associated with DBS. Lack of awareness of what DBS treatment is, as well as its distinction from electroconvulsive therapy, has been shown to influence public attitudes and views about it when considering it as a potential treatment option [26,28,29,53].

Part of the perspective on whether DBS is acceptable for people with OUD is dependent on whether addiction is considered a mental disorder, a theme that clearly intersects with stigma. The medical field has classified OUD as a mental health disorder [60], but that does not mean that society sees it that way. If the public does not agree that OUD is a mental disorder, they are less likely to agree with supplying treatments that are more costly (such as DBS) [61,62], as we saw with comments coded under “Is Addiction a Mental Disorder?” These overlapping topics feed into the comments coded under “Resource Allocation” and “Medical Professional Issues.” For example, similar to a previous publication [46], comments under the “Resource Allocation” theme focused on access to health care as a topic of conversation, with several comments shaped by whether or not psychiatric disorders are considered medical conditions. Likewise, past abuses by the medical profession with psychiatric interventions resonate in this analysis and previous work examining psychiatric interventions.

For the themes that appeared more frequently in comments on one of the two stories, it is possible that the framing of each story shaped the relevance of certain themes for comments in one but not the other. For the 2019 story, significantly more comments were coded with the themes “Controversial,” “Scientific Validity,” and “Cautionary Realism.” A previous study examining psychiatric neurosurgical interventions, including DBS [46], found a similarly large number of comments coded under these themes. It is possible that the experimental nature of DBS in psychiatry shapes these comments. Interventions directly affecting the brain are not without controversy, however, the difference in frequency of this theme between 2019 and 2021 could be the result of increasing awareness of DBS for other disorders, and as such it was not viewed as being as controversial for the later story. Readers of the 2019 story may have been skeptical that refractory OUD could be treated at all and surprised that the patient was willing to participate in this clinical trial, which would be why so many comments were coded as “Cautionary Realism.” By contrast, the second story provided the anecdotal story of the patient having had success with DBS, giving “Scientific Validity” to the procedure; this framing might have alleviated some of the hesitancies about using this neuromodulating technology reflected in comments referring to the 2019 story.

“Overall Therapeutic Need,” “Overall Social Issues,” and “Personal Anecdote” were major themes in the 2021 story. While it is the case that not all treatments work for all patients [20,23], for OUD in particular there has been a clear need to find effective alternatives [63]. The theme of “Overall Therapeutic Need” may have been more prevalent for the 2021 story because commenters raised awareness about the lack of effective treatments for OUD. The description of the FDA’s requirements for enrollment into this clinical trial in the 2021 story, as well as the title, may have contributed to this theme [38]. In addition, “Therapeutic Need,” a prevalent theme in previous studies examining psychiatric neurosurgical interventions [46], resonates with the scientific push to find novel treatments for refractory psychiatric conditions. “Overall Social Issues” reflected the complexity of social issues entangled with the application of neurotechnology as a treatment option for psychiatric disorders. Finally, as we have seen with previous news comments analysis [46,64], readers take the opportunity to share their own stories related to the topics covered in the story giving context to their perspectives on both the disorder and the treatment at hand. This makes the theme “Personal Anecdote” particularly helpful in hearing the voices of those who have been affected by OUD or treated with DBS.

### Limitations

This study was limited by the number of comments that exist in response to each story, and by the fact that only 2 stories were available to analyze. In addition, the comments reflect the opinions of readers of web-based stories in newspapers, such as the Washington Post, and more specifically, those who choose to comment on the story. As such, they may not be representative of the concerns of other groups. Comment analysis also has an inherent limitation related to the interpretability of comments with a limited amount of context. Because the data was anonymized and commenters only used usernames, the analysis did not take into consideration potential commenters’ access to clinical information, demographics, or socioeconomic status. In addition, one commenter could comment several times with a similar perspective on the topic, which could have skewed the frequency of themes.

### Conclusions

Comments containing the perspectives and attitudes of society on DBS as a treatment method for OUD elucidated themes that include sociopolitical issues, positions on the use of technology, and technological and scientific issues. Future work may include further exploration of the public’s perception of the use of DBS as a novel treatment for OUD, as well as other key public views (including patients) on the use of DBS as a potential treatment method for OUD.

## Abbreviations

DBS: deep brain stimulation
*DSM-5*: *Diagnostic and Statistical Manual of Mental Disorders (Fifth Edition)*
FDA: Food and Drug Administration
IRB: institutional review board
OUD: opioid use disorder

## Data Availability

The data sets generated during and analyzed during this study are based on publicly available data.
